# High Performance Thin Layer Chromatographic Method for Simultaneous Estimation of Ibuprofen and Pseudoephedrine Hydrochloride

**DOI:** 10.4103/0250-474X.43018

**Published:** 2008

**Authors:** S. S. Chitlange, D. M. Sakarkar, S. B. Wankhede, S. G. Wadodkar

**Affiliations:** Pad. Dr. D. Y. Patil Institute of Pharmaceutical Sciences and Research, Sant Tukaram Nagar, Pimpri, Pune- 411 018, India; 1 S. N. Institute of Pharmacy, Pusad-445 204, India; 2 Smt. Kishoritai Bhoyar College of Pharmacy, Kamptee, Nagpur-441 002, India

**Keywords:** Ibuprofen, pseudoephedrine hydrochloride, HPTLC, t-butanol, ethyl acetate, glacial acetic acid, water

## Abstract

High performance thin layer chromatographic method is developed for simultaneous estimation of ibuprofen and pseudoephedrine hydrochloride in tablets. Silica gel 60F_254_ plates were used as stationary phase and t.butanol: ethyl acetate: glacial acetic acid: water (7:4:2:2 v/v) as mobile phase. Wavelength selected for analysis was 254 nm. Percent estimation of ibuprofen and pseudoephedrine hydrochloride was found to be 99.56% and 98.77%, respectively. Percent recovery for both the drugs was found in the range of 98.27% to 100.91%, respectively.

Ibuprofen (IBU) is a non steroidal antiinflammatory agent with propionic acid group. Chemically it is (RS)-2-(4-isobutyl phenyl) propionic acid. Pseudoephedrine hydrochloride (PEH) is a sympathomimetic agent. Chemically it is (1S,2S)-2-methyl amino-1-phenyl-1-propanol hydrochloride. Fixed dose combination tablet containing IBU (200 mg) and PEH (30 mg) is available for clinical use. IBU is official in IP, BP and USP. PEH is official in IP, BP, EP and USP. Literature survey revealed spectrophotometric^1^, spectrofluorometric^2^, HPLC^3^–^4^, GC^5^ and HPTLC^6^ methods for estimation of IBU alone or in combination with other drugs, in pharmaceutical formulation and biological fluids. PEH is reported to be estimated by non-aqueous titrimetry^7^, derivative spectrophotometry^8^, HPLC^9^,^10^, capillary electrophoresis^11^,^12^ individually or in combination with other drugs, in pharmaceutical formulation and biological fluids. Spectrophotometric^13^,^14^ methods are reported for simultaneous estimation of these drugs in tablet formulation. In the present work a successful attempt has been made to estimate both these drugs simultaneously by economical and less time consuming HPTLC method.

A Camag-HPTLC system comprising of Linomat-IV automatic sample applicator and Camag TLC scanner 3 with CAT’S version 4.0 software were used for sample application and quantitative evaluation respectively. Samples were applied as bands (band size: 6 mm at 6 mm interval) under a stream of nitrogen on aluminium plates coated with silica gel 60F254 (10×10 cm, Merck) and chromatographed using tertiary butanol:ethyl acetate:glacial acetic acid: water (7:4:2:2 v/v) as mobile phase. Ascending development was performed in a saturated twin-trough TLC chamber. Chromatogram was evaluated by scanning in absorbance/reflectance mode at 254 nm using slit dimensions 4×0.45 mm and quantitation was done by comparing peak height of standard and sample peaks.

Standard solution containing 8 mg/ml of IBU and 1.2 mg/ml of PEH was prepared in methanol. To study the linearity of detector response, the standard stock solutions were appropriately diluted and accurately measured volume ranging from 1 to 10 μl was applied on the TLC plate. The plate was then chromatographed in the selected chromatographic conditions. A calibration graph was constructed by plotting peak height versus concentration.

For estimation of IBU and PEH in tablets, an accurately weighed quantity of tablet powder equivalent to 200 mg of IBU was transferred to 25 ml volumetric flask, shaken with 10 ml of methanol for 15 min. and the volume was then adjusted to the mark with methanol. The solution was then filtered through whatman Grade I filter paper and 6 μl of the filtrate (six bands) and standard solution (one band) was applied on the TLC plate and chromatographed. Amount of both the drugs were estimated by comparing the peak height of standard and sample bands. The results are shown in Table 1 and respective densitogram is shown in fig. 1.

**TABLE 1:** RESULTS OF ESTIMATION IN TABLET AND RECOVERY STUDIES

Sample	Label claim (mg/tablet)	% Label claim*, SD, CV		% Recovery*, SD, CV	
			
		IBU	PEH	IBU	PEH
Standard laboratory mixture	-	99.01±0.889	99.46±0.731	-	-
		0.030	0.024		
Arinac tablet	IBU 200	99.56±1.668	98.77±1.452	100.69±1.662	101.03±1.854
	PEH - 30	0.016	0.024	0.016	0.018

* Indicates mean of five observations, ± indicates standard deviation. IBU stands for ibuprofen and PEH denotes pseudoephedrine hydrochloride.

**Fig. 1:**
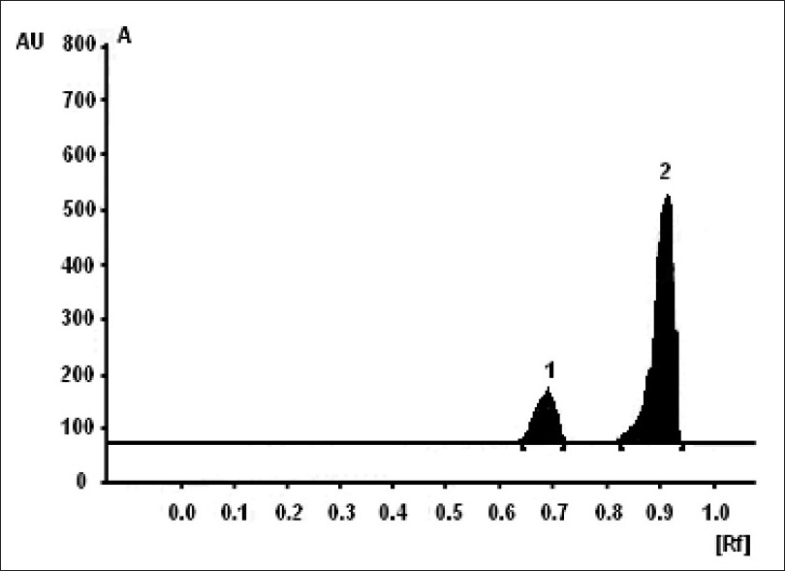
Chromatogram of marketed formulation. 1- pseudoephedrine hydrochloride and 2- ibuprofen

To study the accuracy of the proposed method recovery studies were carried out using standard addition method. The percent recovery was calculated by using the formula, % recovery= (T-A)/S×100, where T is total amount of drug estimated, A is the amount of drug contributed by tablet powder and S is the amount of pure drug added. Result of recovery studies are shown in Table 1.

The system repeatability was studied by applying five replicate applications of standard solution on TLC plate. The plate was chromatographed and the standard deviation for peak height of IBU and PEH was calculated. The robustness of the method was evaluated by studying analyst-to-analyst, intra and inter day variations.

The selected chromatographic conditions were found to effectively separate IBU (Rf = 0.91) and PEH (Rf = 0.68). The linearity for detector response was observed in the range of 45.6-75.6 μg for IBU (correlation coefficient = 0.9934) and 6.8-11.3 μg for PEH (correlation coefficient = 0.9963). Percent amount of IBU and PEH estimated in the average weight of tablet were found to be 99.56% (standard deviation = ±0.1.668) and 98.77% (standard deviation = ±1.452), respectively. The low values of standard deviation indicate the precision of the method. Percent recovery for IBU and PEH was found to be 100.69% (standard deviation = ±1.662) and 101.03% (standard deviation = ±1.854) indicating that the excipients does not have interference in their estimation. The system repeatability studied by five replicate applications of standard solution. The standard deviations for peak height were found to be ±0.11 for IBU and ±0.06 for PEH. The standard deviation for robustness studies was below 2%.

Based on the above results it can be concluded that the proposed HPTLC method is accurate, precise, specific and reproducible. The method is also economical and less time consuming and can be used for routine analysis of ibuprofen and Pseudoephedrine hydrochloride.

## References

[1] Wahbi AA, Hassan E, Hamdy D, Khamis E’ Barary M (2005). Spectrophotometric methods for the determination of ibuprofen in tablets. Pak J Pharm Sci.

[2] Damiani PC, Bearzotti M, Cabazon MA (2001). Spectrofluorometric determination of ibuprofen in pharmaceutical formulation. J Pharm Biomed Anal.

[3] Farrar H, Letzig L, Gill MJ (2002). Validation of a liquid chromatographic method for the determination of ibuprofen in human plasma. Chromatogr B Analyt Technol Biomed Life Sci.

[4] Wang P, Qi M, Liu L, Fang L (2005). Determination of ibuprofen in dog plasma by liquid chromatography. J Pharm Biomed Anal.

[5] Paik MJ, Kim KR (2004). Optical purity determination of ibuprofen in tablets by achiral gas chromatography. Arch Pharm Res.

[6] Save TK, Parmar DV, Devaranjan PV (1997). High Performance thin layer chromatographic determination of ibuprofen in plasma. J Chromatogr B Biomed Sci Appl.

[7] (1996). Indian Pharmacopoeia.

[8] Mabrouk MM, EI-Fatatry HM, Hammad S, Wahbi AA (1998). Simultaneous determination of loratadine and Pseudoephedrine in pharmaceutical formulation by RP-LC and derivative spectrophotometry. Se Pu.

[9] Ge OH, Zhou Z, Zhi XJ, Wang H (2001). Simultaneous determination of Pseudoephedrine and chlorpheniramine in human plasma by HPLC-UV detection method. Se Pu.

[10] Dinc E, Ozdemir A, Aksoy H, Ustundag O, Baleanu D (2003). Chemometric determination of naproxen sodium and Pseudoephedrine hydrochloride in tablets by HPLC. J Pharm Biomed Anal.

[11] Dong Y, Chen X, Chen Y, Hu Z (2005). Seperation and determination of Pseudoephedrine, dextromethorphan, diphenhydramine and chlorpheniramine in cold medicines by nonaqueous capillary electrophoresis. J Pharm Biomed Anal.

[12] Chen H, Chen X, Pu Q, Hu Z, Zhao Z, Hooper M (2003). Seperation and determination of ephedrine and Pseudoephedrine by combination of flow injection with capillary electrophoresis. J Chromatogr Sci.

[13] Palabiyik I. M, Dinc E, Onur F (2004). Simultaneous spectrophotometric determination of Pseudoephedrine hydrochloride and ibuprofen in a pharmaceutical preparation using ratio spectra derivative Spectrophotometry and multivariate calibration techniques. J Pharm Biomed Anal.

[14] Ivanovic D, Medenica M, Markovic S, Mandic G (2000). Second-derivative spectrophotometric assay of Pseudoephedrine, ibuprofen and loratadine in pharmaceuticals. Arzneimittel Forschung.

